# Effect of a patient-led educational session on pre-clerkship students’ learning of professional values and on their professional development

**DOI:** 10.1080/10872981.2020.1801174

**Published:** 2020-07-30

**Authors:** Lavjay Butani, Colleen Sweeney, Jennifer Plant

**Affiliations:** a Department of Pediatrics, University of California Davis Medical Center, Sacramento, CA, USA; b Department of Biochemistry and Molecular Medicine, University of California Davis School of Medicine, Sacramento, CA, USA

**Keywords:** Patient panels, pre-clerkship education, professionalism, professional identity formation, reflective practice

## Abstract

**Background:**

While there are several curricula using patients as educators, little has been published on how they affect student learning and professional development.

**Objective:**

To explore what 1^st^ year medical students learn about professional values from a patient-led educational experience and how it affects their professional development.

**Design:**

We piloted a pediatric patient and family-led educational session during the molecular medicine course, with the goal of sharing the experience of caring for a child with a chronic illness. Following the session, students were required to submit a written reflection on what they learned and the impact the session had on them. All reflections from one academic year were qualitatively analyzed by two investigators and organized using HyperRESEARCH software. A content analysis approach was used to generate codes and emergent themes. Two theoretical lenses guided the analyses: Arnold’s framework on professional values and the lens of professional identity formation, described as a process by which health care professionals “think, act and feel like a physician.

**Results:**

Students gained an appreciation of professional values, especially humanism and excellence, and how clinician role models reinforce these values. Reflective writings demonstrated recognition among learners that their identity involved being active participants in health care delivery and not just as passive classroom learners. Students were motivated to study diligently and be patient advocates; some questioned their skills in dealing with ambiguity and with the health-care system, resulting in a sense of helplessness.

**Conclusion:**

Students learn the importance of professional attributes and of clinician role models through a pediatric family teaching experience. They are motivated, displaying glimpses of their future role as caregivers and patient advocates; however, some also express fear and doubt their own abilities. Based on this, a debriefing session has been introduced to prevent a negative effect on learner self-efficacy.

## Introduction

It has long been recognized that early and direct contact of learners in the health-care professions with patients (patient-centered learning) is critical to the development of higher level competencies such as professionalism and communication skills. [1] Several potential benefits arise from learning activities involving patients. These include more robust development of illness scripts by promoting contextual learning, greater motivation to learn by creating an authentic environment, [2] building skills in diagnostic reasoning, communication and patient interaction [3] and heightening empathy by bearing witness to patients’ narratives and stories, [4] all of which can contribute to professional identity formation (PIF).

Patients have played different educational roles in a variety of settings, as comprehensively reviewed by Towle et al. [5] Educational partnerships with patients have ranged from brief clinical encounters with volunteer/standardized patients to teach and/or evaluate specific clinical skills, [6,7] discussion panels where patients share their stories, recurring home visits with patients experiencing unique and challenging health-care needs, [8] and even patient involvement in curriculum development. [9] There are limited published data on the effect of shared educational experiences with patients/families and students in the health-professions, especially in the pre-clerkship medical education curriculum.

Driven by several factors- the limited pediatric content in the pre-clerkship curriculum at our institution (mirroring national data) [10], the leadership of an innovative course director [CS] for the 1^st^ year molecular medicine course and a desire to engage medical students in a curricular block that is motivationally challenging due to the complex biochemical pathways covered- we introduced a 1-hour educational session led by a pediatric family. The goal of the session was to expose students to the manifestations and lived experience of patients with a chronic illness (which in this case just happened to be a metabolic disorder), broadening the transferability of their learning from the session. The specific learner outcome objectives were for the students to be able to 1) explain the signs and symptoms of cystinosis based on the pathophysiology of the metabolic abnormality, 2) discuss the pros and cons of regenerative medicine as a solution to metabolic diseases, and 3) critically reflect on the impact of a chronic illness on various stakeholders. As part of the session, students were expected to pre-read an article on the underlying biochemical pathway and its perturbations that lead to the clinical manifestations of cystinosis. During the mandatory on-site session, the patients (twin siblings with cystinosis) and their mother were introduced to the students by their treating provider [LB], who discussed how the children presented and were diagnosed and the provider’s experience in being a part of their care. Following this, the family shared its perspective on the health-care system and the impact of illness on their lives. The majority of the time was spent in an unstructured question and answer session led by the students (directed towards the patients/family and/or their health-care provider). Following the session, all students were required to submit a 1-page written reflection; the formatting of the submission (length, word count, spacing etc.) was not proscribed. The reflective prompt was for the students to use varying perspectives (the patients’, the family’s, the health care profession’s, the learner’s etc.) and ‘reflect on the experience of living with a chronic metabolic condition or taking care of/learning from patients with a chronic metabolic condition.’ The reflective writings were required but were not evaluated for purposes of learner summative assessment.

Reading through the reflective writings from our assignment, the authors noted rich learning pertaining to both the transmission of professional values and to students’ explorations of what it means to be a health care provider responsible for partnering in the care of patients with chronic medical conditions. Based on this observation, we proposed this study, with the aims of exploring student learning about professional values and the effect of the session on their professional identity. Two theoretical frames were used to guide the qualitative analyses. The first, Arnold and Stern’s framework on professional values and behaviors, [11] conceptualizes professionalism as a virtue towards which all physicians continuously strive; according to them, professionalism is demonstrated via behaviors that encompass clinical competence, effective communication skills and ethical and legal understanding of the profession. The four aspirational values that drive these behaviors are altruism, accountability, humanism and excellence. [11]The second guiding theoretical frame drew from the published literature on professional identity formation, the essence of which is eloquently captured as the process by which learners come of ‘think, act and feel like a physician.’ [12,13] The research team acknowledged up front that a single educational session was highly unlikely to have a sustained impact on students’ identity formation. Nevertheless, we felt that the study, as conceptualized, would provide glimpses into the thought process and emotions of students related to how they ‘think, act and feel like a physician,’ after our educational session and that this was therefore worthy of investigating.

## Materials and methods

All 99 de-identified written narratives from the 2017–2018 academic year were included in the analysis. The mean and median word count for the narratives were 444.6 and 442 words respectively. Two investigators [LB, JP] reviewed each narrative and independently coded them line-by-line. When addressing the first aim, we used a directed content analysis approach [14] and referred to the previously mentioned framework of professionalism presented by Arnold and Stern. ^11^ We coded for student learning pertaining to one or more of the four aspirational professional values of altruism, accountability, humanism and excellence. For the second aim of the study, we used a conventional content analysis approach. [14] Narratives were read closely by the two investigators, each of whom made notes which led to the emergence of codes pertaining to how learners come of ‘think, act and feel like a physician.’

The investigators met on a recurring basis, initially to review and reconcile codes and subsequently to develop sub-themes and themes through the process of analyzing and sorting codes into broad thematic categories. Differences were reconciled by consensus during face to face meetings. The third investigator [CS] reviewed the coding process to ensure trustworthiness. Since this was a retrospective data analysis, we elected to analyze all reflective writings and not stop our analysis based on thematic saturation.

To increase the trustworthiness of our findings, we explicitly discussed our own biases to promote reflexivity and address the impact of our personal beliefs on the analytic process. We maintained an audit trail of all analyses and coding discussions and organized our data using HyperRESEARCH Version 3.7.3 (ResearchWare Inc, Randolph, MA). The study was approved by the Institutional Review Board of the University of California Davis and granted an exempt status.

## Results

### Learning about professional values

Students gained a heightened appreciation of all four professional values as described in Arnold and Stern’s model, [11] with a predominant emphasis on *humanism* and *excellence* (See Table 1 for representative quotes). Related to humanism, hearing the family’s stories and voices encouraged learners to consider *seeing the whole person and not just the disease* as well as to appreciate the importance of *providing family centered care* and of *empathy and compassion*. One student wrote: *‘As a member of the health care community, I immediately felt the default emotion we all feel when people tell us that they are sick: sympathy. However, I could feel that gap slowly closing as the family told their story from not only a patient perspective, but almost that of a familiar friend.’ Excellence*, as a value, included elements of *duty towards patients, research as a means to improve patient care, importance of competence and lifelong learning, acknowledging limitations of science and of health care providers* and *team work*. A representative quote pertaining to *research as a means to improve patient care* was: ***‘****It has motivated me to work that much harder at my studies and to strive to pursue research so that issues such as cystinosis can be a thing of the past in the future. I have high hopes for the future as my fellow peers and I move forward.’* A few learner reflections also touched upon how the session instilled in them a sense of *accountability* towards communities and of *altruism*.

**Table 1.** Student learning pertaining to professional values.

Professional value	Representative Quotes
***Humanism*** *-Seeing the whole person and not just the disease* *-Providing family centered care* *-Empathy and compassion*	*‘I am grateful that they were able to present their story to us and give all of us medical students an ability to actually put faces to certain diagnosis. I feel as if while in medical school we start to forget sometimes the significant impact these diseases have on individual lives’*
***Excellence*** *-Duty towards patients* *-Research as a means to improve patient care* *-Competence and lifelong learning* *-Acknowledging limitations of science and of health care providers* *-Team work*	*‘After reflecting more on this case and accepting that I will not ever know everything, I realized that perhaps as a physician, my most important role will be to continue to fight for my patient if I know something is wrong, even if I don’t know the answer, because someone will and that persistence is what will save a child’s life’*
***Accountability***	*‘Learning about this orphan disease, how many lives are still affected had us thinking that these should not be ignored and that we should start doing something about this. We were talking about the possibility of starting a student interest group on campus to increase awareness of these different orphan diseases’*
***Altruism***	*‘I aspire to serve my patients and carry this burden, so they don’t have to and, so they can rely on someone during their periods of isolation and health crisis’*

### Impact on student identity

Student reflective writings demonstrated a keen awareness among learners about the importance of them becoming *more legitimate participants in health care* provision as opposed to being solely imbibers of medical knowledge (see Table 2). This was evidenced via their acknowledgement of the *importance of pre-clerkship foundational learning* in being able to provide excellent care, the *benefits of contextual learning* (in solidifying content knowledge and in seeing the patient as more than a disease), the realization of the *ambiguity in medical practice*, the need for *balancing their naïve idealism with the realities* of our resource-limited health care environment and the *duty/obligation that providers have towards patients*. A representative quote that illustrates many of the above was: *‘Although the clinical correlate case was only an hour long, I was able to learn about the presentation of a specific genetic disease, utilize my knowledge of renal physiology to understand the symptom manifestations, and learn more about the patient health perspective and experience. The family gave me insight on what it means to live with a serious genetic disease and taught me that medicine does not end with just diagnosis. Rather, continuing care, patient advocacy, and continuing research are important features of being a doctor.’*

**Table 2.** Themes and subthemes related to the effect of the patient-led educational session on students’ professional identity.

Themes	Subthemes	*Representative Quotes*
*Becoming legitimate participants in caring for patients*	*-The importance of pre-clerkship foundational learning* *-Benefits of contextual learning* *-Ambiguity in medical practice* *-Balancing idealism with reality* *-Duty towards patients* *- Importance of clinician role models*	*‘There are many times as a medical student that I feel overwhelmed by the wealth of material we learn each and every day. However, being able to hear the xxx family’s story reminded me why everything we learn is so critically important to our future caregiving as physicians and health care providers.’* *‘Although it is impossible to have all the possible differentials at your fingertips at all times, the thoroughness of Dr.xxx’s work with the xxx family gives me something to strive for within my medical career’*
*Variable personal reactions*	-Motivation and inspiration to grow and/or advocate	*‘From their stories, I hope to remain receptive to feedback and appreciative of when my patients engage me in my decision making so I can, not only remain vigilant, but also involve them in their own care and treatment plan, which has been shown to improve patient outcomes’*
	*-Questioning without commitment*	*‘One of the questions I have always had is how to tell patients that their physician has no idea what is going on with them. It is difficult for us medical students to understand the balance the desire to not lose the faith of our patients and admitting that we do not have all the answers’*
	*-Sense of helplessness*	*‘Even in our doctoring classes, the discussion is constrained to things that we learned recently, and the course coordinator has a piece of paper with the answer. At least he or she knows the answer we are supposed to reach. The fact that it will not always be so in the real world, is a scary thought’*

Students also recognized the *importance of clinician role models* in both transmitting professional values and helping promote professional identity development. Reflecting on the family’s interactions with a myriad of health-care professionals, most of whom inspired the family’s confidence and gratitude, learners appreciated the value of role models in reinforcing the impact professional values have on patient care: *‘When I reflect on the cystinosis panel, one of the first things that I remember is the unique relationship between the family and their doctor. His name escapes me as I write this, but he was there and very present, playing a supportive yet key role in the presentation of this family’s struggles and joys. I cannot help but wonder what exactly is the role of this doctor in the lives of the family, outside of this presentation. When the dramatic symptoms of an illness like cystinosis become a routine occurrence, so must the relationship between family and doctor become more interwoven. I imagine the doctor to be almost a member of the family. Decisions are made by the parents and their doctor.’*

The second theme encompassed the spectrum of *variable personal reactions* noted in the assignments. Three sub-themes were noted. Student reflections, in general, demonstrated evidence that the students were *motivated and inspired* to push themselves to grow and be advocates for patients. However, much to our surprise, learners also *questioned their own skills and abilities* in being able to deal with the inherent ambiguity of medicine and/or with health care inequities without a commitment to becoming change agents. Some reflections went further and expressed a *sense of helplessness* without an articulated plan on how to move forward and address the challenge, as illustrated by the following quote: *‘I began to worry about my competencies in the field and whether I would be prepared to deal with rare cases like this. Now as I reflect, I realize I was struck with fear. The fear of not knowing everything and missing a diagnosis. I kept thinking of the family, and how for many months they lived in this grey area of not knowing what was happening to their children.’*

None of the reflections noted negative comments about the educational experience itself.

## Discussion

The transition from the pre-clerkship to clerkship curriculum is a particularly challenging one for medical students, stemming from a variety of factors such as working in a chaotic, unstructured and new clinical environment, dealing with the uncertainty of medicine, and having difficulty in applying pre-clerkship theoretical knowledge to a clinical context. One of the many benefits of clinical experiences with real patients early in the pre-clerkship years is to help with this transition. This need for early exposure is even greater with pediatric patients and families, since children have unique needs and require a different skill set on the part of learners and health care providers. Medical students, in general, feel ill prepared to interact with children during the clerkships due to the paucity of such early pediatric-focused clinical experiences. [10] We introduced our innovative curriculum to bridge this gap.

Patient-led learning experiences are infrequently described in the literature; [15] even when such experiences are incorporated into curricula, their impact on student learning and growth remains understudied. The few published reports of educational partnerships with pediatric patients and their families describe curricula that are either time/resource intensive and logistically challenging, [9,16] directed towards more advanced learners such as pediatric residents [8,9] or narrowly focused on a specific content areas, most commonly health-care needs of children with developmental disabilities. [9,16] To our knowledge there have been no published curricula that have incorporated pediatric patients and families within the context of a medical school pre-clerkship basic science educational session to reinforce ‘textbook learning,’ while at the same time reinforcing the humanistic core of the medical profession. An educational session somewhat similar to ours in its intent was described in a French dental school curriculum, where a mother of a young patient with ectodermal dysplasia gave an impactful 1-hour talk, integrated into the basic science instruction, about her and her child’s experiences with the health-care system. [17] Students reported that this session was interesting, motivating and memorable.

Our teaching session and the qualitative analysis of students’ written reflections after the session add to the existing literature and demonstrate the potential effect of partnering with pediatric patients and their families within the pre-clerkship basic science instruction to bring greater meaning and value to the teaching experience for all involved. Sessions such as ours allow learners to move past the disease and see patients as people, getting a glimpse into their day to day personal lives and their interaction with the health-care system; this can be motivating for learners by providing a contextual experience to otherwise seemingly irrelevant classroom didactic teaching. Not only do they learn to appreciate humanism and excellence as key professional values, they also validate the importance that clinician role models play in inspiring and teaching them about these aspirational professional values as a means to providing competent and compassionate care to patients. Not surprisingly, role modeling is widely recognized as one of the most important strategies for facilitating the development of professional attitudes and behaviors in medical learners. [18] The session also enabled students to see how ‘normal’ of a life, patients with chronic illness can lead and that their disease doesn’t define who they and their families are. The reflective quote from a student, presented above, under *empathy and compassion* describes the shift from feeling sympathy towards the patients at the start of the session, to a more holistic recognition of their humanity.

Following this session, our students expressed an appreciation of the value of building their foundational knowledge base in order to provide competent care and displayed an awareness of the importance of thinking about themselves not just as pure ‘learners’ of medical content, rather as caregivers for patients including potential roles as advocates for patients within the health care system and as biomedical researchers. This progression is a known developmental challenge in professional identity formation. Exposure to clinician role models and reflective writing, two pedagogical strategies thought to help students make this transition [12] were employed in our innovation. Early exposure to patients and families in a clinical setting is another potential pedagogical strategy; [19] our work supports that such early exposure to patients and families in a classroom setting also facilitate identity formation.

While most students were motivated by this educational experience to keep pushing themselves and work with our flawed health-care system, some remained ambivalent about the system and also insecure about their own abilities. Whether this was a byproduct of the unique and rare chronic illness that was the focal point around which the session was built, or a natural consequence of being exposed to the complexities involved in patient care and diagnostic skills at an early stage of training and in a ‘one off’ manner, remains to be determined.

We acknowledge that our study has many limitations. Most importantly, we recognize that a single one-hour educational session is highly unlikely, by itself, to have a sustained or dramatic effect on learning and especially on identity formation. However, sessions such as ours can be easily incorporated into additional pre-clerkship courses, irrespective of the content-focus of the course, to reinforce learning. This type of session aims to provide a broader understanding of the patient experience living with a chronic illness and navigating the health-care system, both of which have common elements across a wide spectrum of chronic illnesses. Moreover, even though the reflective assignment was not summatively evaluated, the required nature of the submission could have affected how students approached the assignment and what they wrote in their narrative. Nevertheless, the experience presented here reinforces the importance of early exposure of medical students to patients and families and this case study highlights the potential benefits of patients and patient panels in helping promote student learning and growth and providing a more holistic context to their pre-clerkship education. Lastly, all three authors are passionate advocates of contextual learning, the humanistic foundations of the practice of medicine, and of reflective practice. Our biases and passion could have positively influenced the way the students received this session. In order to promote reflexivity, we discussed these biases during the data analyses and made a conscious effort to accurately interpret the data.

Based on our experience with this pilot curriculum, additional pediatric family and patient experiences are being introduced into the pre-clerkship curriculum; moreover, a debriefing session is planned to follow these sessions, in order to help re-ground students and minimize a negative effect on their self-efficacy. These will undoubtedly provide additional opportunities to study the impact of patient-panels on student learning and growth.
